# Four-Jointed Modulates Growth and Planar Polarity by Reducing the Affinity of Dachsous for Fat

**DOI:** 10.1016/j.cub.2010.03.056

**Published:** 2010-05-11

**Authors:** Amy L. Brittle, Ada Repiso, José Casal, Peter A. Lawrence, David Strutt

**Affiliations:** 1 Medical Research Council (MRC) Centre for Developmental and Biomedical Genetics and Department of Biomedical Science, University of Sheffield, Sheffield S10 2TN, UK; 2 MRC Laboratory of Molecular Biology, Hills Road, Cambridge CB2 0QH, UK; 3 Department of Zoology, University of Cambridge, Downing Street, Cambridge CB2 3EJ, UK

**Keywords:** CELLBIO, SIGNALING, DEVBIO

## Abstract

The *Drosophila* genes *fat* (*ft*) and *dachsous* (*ds*) encode large atypical cadherins that collaborate to coordinately polarize cells in the plane of the epithelium (planar cell polarity) and to affect growth via the Warts/Hippo pathway [1–3]. Ft and Ds form heterodimeric bridges that convey polarity information from cell to cell [4–7]. *four-jointed* (*fj*) is a modulator of Ft/Ds activity that acts in a graded fashion in the abdomen, eye, and wing [8–11]. Genetic evidence indicates that Fj acts via Ds and/or Ft [4, 6–9, 12], and here we demonstrate that Fj can act independently on Ds and on Ft. It has been reported that Fj has kinase activity and can phosphorylate a subset of cadherin domains of both Ft and Ds in vitro [13]. We have used both cell and in vitro assays to measure binding between Ft and Ds. We find that phosphorylation of Ds reduces its affinity for Ft in both of these assays. By expressing forms of Ds that lack the defined phosphorylation sites or have phosphomimetic amino acids at these positions, we demonstrate that effects of Fj on wing size and planar polarity can be explained by Fj phosphorylating these sites.

## Highlights

► Four-jointed can modify the activity of both Dachsous and Fat in vivo ► Mutation of conserved residues in the Four-jointed kinase domain blocks its activity ► Four-jointed phosphorylates Dachsous at 3 specific sites to reduce its binding to Ft ► Four-jointed affects growth and planar polarity via phosphorylation at these 3 sites

## Results and Discussion

### Genetic Data Indicate that Fj Can Act on Ds and Ft In Vivo

**Figure 1:**
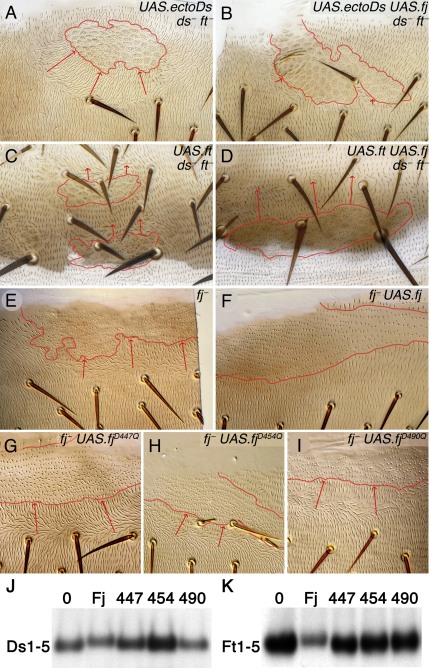
Fj Can Modulate the Activity of Both Ft and Ds In Vivo (A–I) Images of clones (outlined in red) in the dorsal abdomen marked with the hair morphology markers *stc* (A–D) or *pwn* (E–I). Arrows indicate the direction and extent of hair repolarization around the clones. (A) *ds^−^ ft^−^ UAS.ectoDs* clones strongly repolarize hairs behind the clone (100% of clones repolarize with a maximum range of up to seven cells [n = 38]). (B) Repolarization around *ds^−^ ft^−^ UAS.ectoDs UAS.fj* clones is much reduced (48% of clones show some repolarization with a range of one to three cells [n = 48]). (C) *ds^−^ ft^−^ UAS.ft* clones cause repolarization in front of clones (64% of clones repolarize with a maximum range of four cells [n = 25]). (D) Coexpression of *UAS.ft* and *UAS.fj* in *ds^−^ ft*^−^ clones strengthens repolarization (95% of clones repolarized with a maximum range of seven cells [n = 39)). (E) *fj*^−^ clones reverse hair polarity behind the clone (11 of 39 clones, 0 reverse in front). (F) Expression of *UAS.fj* in *fj*^−^ clones rescues the mutant phenotype (and reverses hairs in front of the clone, see [8]), 19 of 25 clones reverse in front, 0 reverse behind; similarly, 21 of 25 *UAS.fj* clones in wild-type flies reverse in front, 0 reverse behind—the comparison suggesting that *UAS.fj* is strongly overexpressed. (G–I) None of the mutated forms of *fj* rescue *fj*^−^, the hairs still being reversed behind the clones upon expression of *UAS.fj^D447Q^* (10 of 28 clones) (G), *UAS.fj^D454Q^* (11 of 39 clones) (H), and *UAS.fj^D490Q^* (12 of 29 clones) (I). (J and K) Truncated forms of Ds and Ft (Ds1-5sec-HA and Ft1-5sec-HA), consisting of the first five cadherin repeats, show reduced mobility on western blots, indicative of modification by phosphorylation [13] when coexpressed with wild-type Fj in D.mel2 cells but not when coexpressed with the three kinase mutants. This mobility shift is reversed by treatment with phosphatase (Figure S1B).

If the levels of Ft, Ds, or Fj are altered in clonal patches of cells, the normal (genetically wild-type) cells nearby have their polarity changed so that, in some cases, all hairs point in toward the clones or, in other cases, outward away from them. Extensive use of this experimental assay has given insight into how these three molecules act to build planar polarity [6–9, 14–16], but it has not been clear whether Fj acts on Ds, on Ft, or on both molecules. We therefore made clones that lacked endogenous Ft and Ds but also overexpressed *ectoDs* (*UAS.ectoDs*), an active form of Ds lacking the intracellular domain [7]. These clones reversed the polarity of hairs behind the clone (Figure 1A), but coexpression of *UAS.fj* with *UAS.ectoDs* largely suppressed this repolarization (Figure 1B). Here, because no Ft was present in the clones, Fj must have inhibited Ds and could not have acted via Ft. Overexpression of *ft* (*UAS.ft*), in the absence of endogenous Ds and Ft, caused a repolarization of hairs so that they pointed away from the clone (Figure 1C), and in this case coexpression of *UAS.fj* increased the range of repolarization (Figure 1D). Therefore, because no Ds was present, Fj must have been acting on Ft itself to promote its activity. Both *ds^−^ ft*^−^ and *ds^−^ ft^−^ UAS.fj* clones do not repolarize the surrounding wild-type hairs [7], arguing that Fj acts by changing the activity of Ds and Ft inside the clones—and not by acting (for example, as a secreted protein) directly on Ds and/or Ft in neighboring wild-type cells. Modification of Ft or Ds inside the clone then presumably alters the strength of Ft-Ds heterophilic interactions across the clone boundary, leading to a change in the range of repolarization around the clone. These experiments demonstrate that Fj can modulate the activity of both Ft and Ds in vivo.

### Point Mutations in Fj Abrogate the Ability to Phosphorylate Ft and Ds

All Fj proteins have a highly conserved region located at the C terminus of the protein (see Figure S1A available online); in the *Drosophila* Fj protein, this domain comprises amino acids 432 to 508. The region shows homology to a kinase-active site [17] containing the essential aspartate residues required for kinase function, as well as other conserved residues nearby. Ishikawa et al. [13] mutated amino acids 490–492 of Fj, which includes the putative Mn^2+^-binding site (D490), and found that this protein could no longer phosphorylate Ft or Ds cadherin domains and was nonfunctional in vivo. We mutated three conserved aspartic acid residues to glutamine at the putative active site (D454Q), the putative Mn^2+^-binding site (D490Q), and at a more N-terminal position (D447Q) within the domain. Mutation of any one of these three sites abolished the protein's ability to phosphorylate Ft and Ds cadherin domains in D.mel2 cells (Figures 1J and 1K; Figure S1B), but these mutant proteins were still bound to Ds and Ft (Figures S1C and S1D) and were localized, like the wild-type protein, in the Golgi (data not shown). Furthermore, unlike the wild-type protein, these mutated molecules were inactive in vivo, either in the presence or in the absence of endogenous wild-type Fj protein (data not shown; Figures 1E–1I). These results argue that the kinase activity of Fj is essential for its function. By coimmunoprecipitation, we detected physical interactions between two regions of Fj and the cadherin repeats 1–5 of both Ft and Ds, again consistent with Fj acting on both molecules (Figures S1E–S1G).

### Fj Inhibits Binding of Ds to Ft

**Figure 2:**
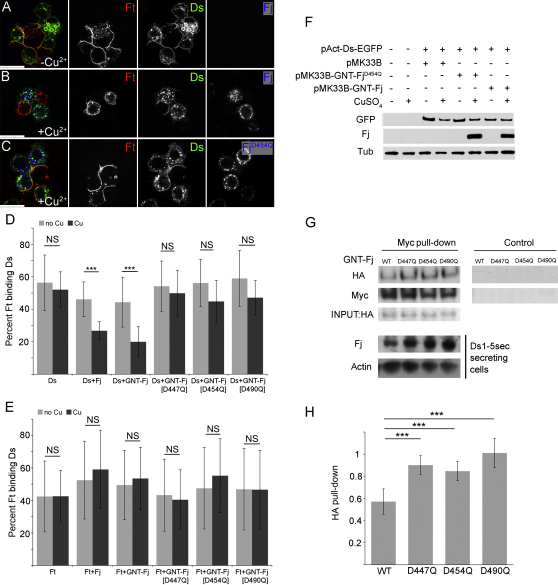
Fj Kinase Activity Inhibits Ds Binding to Ft (A–C) Confocal images showing protein distribution revealed by immunofluorescence of S2 cell aggregates from example binding assays. Cells are labeled for Ft (red), Ds-EGFP (green), and Fj (blue). Scale bar represents 10 μm. S2 cells were doubly transfected with *pAct-ds-EGFP* and *pMK33B-GNT-fj* (A and B) or *pAct-ds-EGFP* and *pMK33B-GNT-Fj^D454Q^* (C) and mixed with cells transfected with *pAct-ft*. Fj expression was induced by addition of 0.14 mM CuSO_4_ (B and C). Ft and Ds bind in neighboring cells, stabilizing each other's localization at the cell surface and forming aggregates (A). GNT-Fj expression appears to reduce the stabilization of Ft and Ds at cell contacts (B), whereas GNT-Fj^D454Q^ expression does not (C). (D and E) Quantifications of Ft-Ds binding in cell aggregation assays. The percent of *ft* cells in the population that bind to *ds* cells was determined. *pAct-ds* (D) or *pAct-ft* (E) was cotransfected with either empty *pMK33B* or *pMK33B* expressing *fj*, *GNT-fj*, *GNT-fj^D447Q^*, *GNT-fj^D454Q^*, or *GNT-fj^D490Q^*, and these cells then mixed with cells singly transfected with either *pAct-ft* (D) or *pAct-ds* (E). Binding of cells in the absence of CuSO_4_ (light gray) was compared to binding in the presence of CuSO_4_ (to induce Fj expression, dark gray) (0.14 mM in D and 0.7 mM in E) to determine whether Fj expression significantly altered binding. Graphs show the mean from five separate experiments; error bars show standard deviation. Student's t tests were applied (^∗∗∗^p < 0.005 between pairs of columns indicated by bars); NS, not significant. (F) Western blot showing Ds-EGFP, Fj, and tubulin levels in S2 cell extracts in the presence or absence of GNT-Fj or GNT-Fj^D454Q^. Note the efficacy of CuSO_4_. (G) Coimmunoprecipitation of Ds1-5sec-HA with Ft1-5-Myc. D.mel2 cells were transfected with Ds1-5sec-HA and either *pMK33B-GNT-Fj* or kinase mutants. Addition of CuSO_4_ (0.14 mM) induced GNT-Fj expression in secreting cells (bottom). Medium containing secreted Ds1-5sec-HA (INPUT:HA) was incubated with cell lysate containing Ft1-5-Myc. Anti-Myc antibodies were used to pull down Ft1-5-Myc, and anti-HA was used to detect Ds1-5sec-HA. Control (right) is a mock immunoprecipitation without Ft1-5-Myc expression. Note that in this experiment, because of variations in transfection efficiency, the Ds1-5sec-HA-secreting cells transfected with wild-type GNT-Fj express a lower level of Fj than those transfected with mutant forms of GNT-Fj; however, even this lower level of kinase-active Fj results in a significant reduction in the amount of Ds1-5sec-HA pulled down. (H) Quantitation of five experiments as shown in (G). Amount of pull-down is shown in arbitrary units. Error bars show standard deviation. Wild-type Fj significantly reduces pull-down of Ds1-5sec as compared to the three kinase mutant forms (p < 0.005). See also Figure S2.

Ft and Ds can form heterodimers, intercellular bridges conveying polarity information from cell to cell [4–7], and we now tested whether phosphorylation of Ds and/or Ft by Fj might regulate this heterodimerization. When *Drosophila* S2 cells were separately transfected with Ft and Ds and then mixed together, they formed cell aggregates; Ds and Ft appeared to stabilize each other's localization at the cell membrane (Figure 2A) [4–6]. We cotransfected *Drosophila* S2 cells with *fj* or *GNT-fj*, a Golgi-tethered form of the protein that is more active in vivo [12], plus either *ft* or *ds*. *fj* was expressed under the control of a Cu^2+^-inducible promoter, whereas *ft* and *ds* were expressed under constitutive actin promoters (Figure S2A). Without the addition of CuSO_4_, no detectable Fj was expressed (Figures 2A and 2F). We then measured the amount of cell aggregation between these doubly transfected cells and cells singly transfected with *ft* or *ds*.

Strong mutual stabilization of Ft and Ds at the interface between cells occurred when no Fj was expressed (Figure 2A). However, when *fj* or *GNT-fj* was coexpressed with *ds*, this mutual stabilization of Ft and Ds was reduced (Figure 2B), and fewer clumps of cells were recorded. To quantify the level of binding between cells, we counted the percentage of Ft cells in the population adhering to Ds cells and compared these data with and without CuSO_4_ (±Fj expression) (Figures 2D and 2E). When *fj* was induced in *ds*-expressing cells, the binding between *ft*- and *ds*-expressing cells was reduced by 40% (Figure 2D). The GNT-Fj form, which is not secreted from cells, was at least as potent as wild-type Fj in inhibiting Ds binding to Ft (Figure 2D), consistent with Fj mediating its effect in the Golgi [12], and not at the cell surface.

No significant effect on Ft-Ds binding was observed when either *fj* or *GNT-fj* was coexpressed with *ft* (Figure 2E). Both the in vivo genetic evidence described above, along with the observation that Ft cadherin domains can be phosphorylated by Fj (Figure 1K; [13]), suggest that Fj might also act on Ft. But if Fj acts on Ft to modulate its binding to Ds, our in vitro assays failed to reveal it.

To determine whether it is the kinase activity of Fj that is responsible for the inhibition of binding, we tested forms of GNT-Fj that were mutated in the kinase domain. When each one of the three mutant Fj proteins was expressed with *ds* in the cell aggregation assay, they, unlike the wild-type protein, had no significant effect on binding (Figure 2D). Furthermore, stabilization of Ft and Ds at the membrane did not seem to be altered by expression of *GNT-fj^D454Q^* (Figure 2C). Therefore, the kinase activity of Fj is required in the Ds-expressing cells to modify Ft-Ds binding.

Because Fj is known to phosphorylate Ds cadherin domains (Figure 1J; [13]), its effects are most likely implemented via changes in the mutual binding affinity of Ft and Ds. But they could also be explained if the level or localization of Ds protein was altered by *fj* expression. To test for this, we expressed *GNT-fj* with *ds-EGFP* and found that Fj does not change the level of Ds-EGFP protein by western blot (Figure 2F), indicating that overall levels of Ds protein are not altered. Importantly, we also determined that the level of Ds that reaches the cell surface was not significantly altered (Figure S2B). These results support the view that it is indeed the binding affinity between Ft and Ds that is modified by Fj.

Finally, we carried out coimmunoprecipitation experiments between fragments of Ds and Ft, known to bind to each other in cell aggregation assays (Figure S2C), and confirmed again that Fj kinase activity modulates Ft-Ds binding. A secreted form of HA-tagged Ds that contains the first five cadherin domains (Ds1-5sec), including amino acid S236, which is phosphorylated by Fj [13], was expressed in cells in the presence of either Golgi-tethered *GNT-fj* or (as controls) forms of *GNT-fj* mutated in the kinase domain. In this assay, expression of *GNT-fj* significantly reduced the amount of Ds1-5sec that was pulled down with Ft1-5 when compared to each of the three *GNT-fj* kinase mutants (Figures 2G and 2H). Hence, Fj phosphorylation of Ds1-5 alters its binding affinity for Ft1-5.

### Mutation of Phosphorylation Sites in Ds Alters Its Ability to Bind to Ft

**Figure 3:**
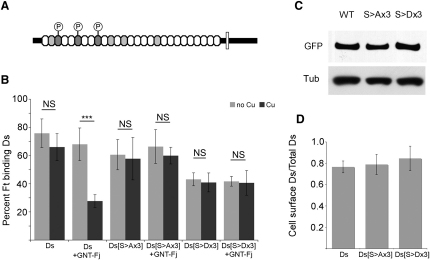
Ds Phosphorylation Sites Mediate the Effects of Fj on Ft-Ds Binding Affinity (A) Diagram representing the structure of Ds protein and localization of phosphorylation sites. Ovals represent the cadherin domains of Ds. Dark gray marks the cadherin domains containing serines that were shown in S2 cells to be phosphorylated by Fj (CAD3 [S236], CAD6 [S561], CAD9 [S881]), and light gray represents cadherin domains containing conserved S/T residues that have not been shown to be phosphorylated by Fj [13]. (B) Cell aggregation assay with Ds phosphorylation mutants. *pAct-ds-EGFP*, *ds^S>Ax3^-EGFP* (S236A, S561A, S881A), or *ds^S>Dx3^-EGFP* (S236D, S561D, S881D) was cotransfected with *pMK33B* empty vector or *pMK33B-GNT-fj*. The percentage of Ft-expressing cells binding to Ds-EGFP-expressing cells was determined. Binding of cells in the absence of CuSO_4_ (light gray) was compared to binding in the presence of 0.14 mM CuSO_4_ (dark gray) to determine whether Fj expression made a significant difference to binding (n = 3, error bars show standard deviation). Student's t tests were applied (^∗∗∗^p < 0.005) and are indicated for each pair of columns (±Cu). In addition, of the first eight columns (four column pairs), only column 4 (Ds + GNT-Fj, +Cu) shows a significant difference from column 1 (Ds, -Cu). The level of binding of Ft to Ds^S>Dx3^-EGFP cells (columns 9 and 11) was significantly lower than to Ds-EGFP (column 1) and Ds^S>Ax3^-EGFP (column 5) (p < 0.005). Note also that the level of binding of Ds^S>Dx3^-EGFP cells (column 9) is not as low as Ds-EGFP when GNT-Fj (column 4) is coexpressed (p < 0.05), suggesting that phosphorylation of the serine residues has a stronger effect on the affinity of binding of Ds to Ft than mutation of these serine residues to aspartate. (C) Western blot comparing levels of Ds-EGFP, Ds^S>Ax3^-EGFP, and Ds^S>Dx3^-EGFP expression in transfected S2 cells. (D) Ratio of cell surface expression versus total levels of Ds-EGFP, Ds^S>Ax3^-EGFP, and Ds^S>Dx3^-EGFP in transfected S2 cells. The levels are not significantly different.

Ishikawa et al. [13] identified three conserved serines (S236, S561, and S881) in Ds cadherin domains that can be phosphorylated by Fj in vitro or in cell-based assays (Figure 3A). We mutated these three serines in *ds-EGFP* to alanine to obviate phosphorylation at these sites (*ds^S>Ax3^-EGFP*). Mutation of these serines did not disrupt the levels of expression or cell-surface localization (Figures 3C and 3D). Nor did it significantly impact on the behavior of the protein: in the absence of *fj* expression, *ds^S>Ax3^-EGFP*-expressing cells can still bind to *ft*-expressing cells at a similar level as do wild-type *ds-EGFP*-expressing cells (Figure 3B). But, in the cell aggregation assay, *ds^S>Ax3^-EGFP*-expressing cells did not respond to coexpression of *GNT-Fj*, unlike control *ds-EGFP*-expressing cells (Figure 3B). These results argue that these three phosphorylation sites are instrumental in the modulation of Ft-Ds binding affinity by Fj.

To make a phosphomimetic form of Ds, we mutated each of these three serines to aspartates so as to add a negative charge, much like phosphorylation itself does (*ds^S>Dx3^-EGFP*). We first confirmed that Ds^S>Dx3^-EGFP protein was expressed at a similar level to Ds-EGFP and Ds^S>Ax3^-EGFP by western blot (Figure 3C) and that the level of protein at the cell surface was similar (Figure 3D). However, we found that *ds^S>Dx3^-EGFP*-expressing cells had a significantly reduced level of binding to Ft cells when compared to wild-type *ds-EGFP*- or *ds^S>Ax3^-EGFP*-expressing cells (Figure 3B), although we noted that the reduction was not as strong as when Ds-EGFP was modified by Fj, suggesting that the S>D substitution is not a perfect phosphomimetic. Nevertheless, consistent with all of our findings, mimicking phosphorylation at these sites reduced binding to Ft. Coexpression of *GNT-Fj* with *ds^S>Dx3^-EGFP* did not reduce binding further (Figure 3B), indicating that if there were additional sites in Ds that could be phosphorylated, they do not contribute significantly to the regulation of Ft-Ds binding.

### Phosphorylation of Ds Is Relevant In Vivo

To investigate the importance of the Ds phosphorylation sites in vivo, we assayed the activity of the Ds^S>Ax3^-EGFP and Ds^S>Dx3^-EGFP proteins in flies. We generated transgenic flies that expressed *ds^S>Ax3^-EGFP* and *ds^S>Dx3^-EGFP*, as well as wild-type *ds-EGFP*, under the control of the *Act5C* promoter. To ensure that all forms of Ds were expressed at the same level, we used site-specific integration to insert the transgenes at the same chromosomal location.

**Figure 4:**
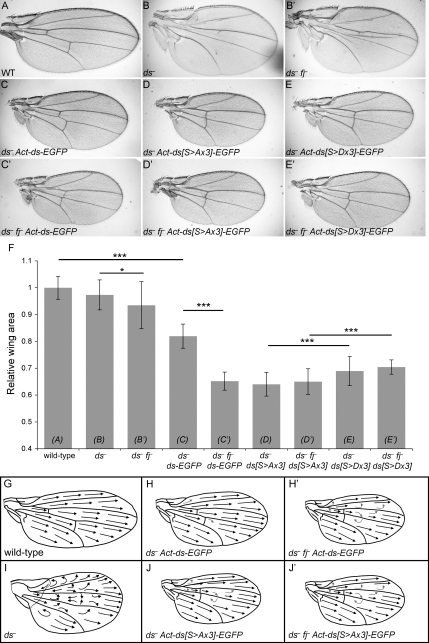
Ds Phosphorylation Sites Are Required In Vivo for Fj to Modulate Growth and Planar Polarity (A–E′) Male adult wings. In (C)–(E′), all genotypes include *Ubx-flp* to activate expression under control of the *Act5C* promoter of transgenes cloned into the vector *pAttB-Act-FRT-polyA-FRT*. All images are of the same magnification. To help with interpretation, we indicated removal of *fj* simply as instanced here: (B) is one genotype and (B′) is the same genotype without *fj*. (A) Wild-type. (B) *ds^UA071^/ds^38k^*. (B′) *ds^UA071^ fj^d1^ /ds^38k^ fj^P1^*. (C) *ds^UA071^/ds^38k^; Act-ds-EGFP/+*. (C′) *ds^UA071^ fj^d1^ /ds^38k^ fj^P1^; Act-ds-EGFP/+*. (D) *ds^UA071^/ds^38k^; Act-ds^S>Ax3^-EGFP/+*. (D′) *ds^UA071^ fj^d1^ /ds^38k^ fj^P1^; Act-ds^S>Ax3^-EGFP/+*. (E) *ds^UA071^/ds^38k^; Act-ds^S>Dx3^-EGFP/+*. (E′) *ds^UA071^ fj^d1^ /ds^38k^ fj^P1^; Act-ds^S>Dx3^-EGFP/+*. (F) Quantitation of wing blade area, relative to wild-type. Twenty wings from male flies were measured per genotype; error bars show standard deviation. Genotypes can be compared directly with wings pictured above (A–E′ written in columns). Student's t tests were applied (^∗∗∗^p < 0.005, comparisons between columns linked by bars). For (B) (*ds*^−^) and (B′) (*ds*^−^*fj*^−^), a small difference in wing size was found (^∗^p < 0.05) only after measuring 40 wings. This difference in size suggests that *fj* can have a small effect on wing size even in the absence of *ds* activity, although the mechanisms are unclear. (G–J′) Planar polarity is shown by the direction of hairs on the ventral surface in adult wings. Arrows indicate the direction hairs point. Light gray arrows indicate swirls that are not found in wild-type wings. (G) Wild-type. (H) *ds^UA071^/ds^38k^; Act-ds-EGFP/+*. (H′) *ds^UA071^ fj^d1^/ds^38k^ fj^P1^; Act-ds-EGFP/+*. (I) *ds^UA071^/ds^38k^*. (J) *ds^UA071^/ds^38k^; Act-ds^S>Ax3^-EGFP/+*. (J′) *ds^UA071^ fj^d1^/ds^38k^ fj^P1^; Act-ds^S>Ax3^-EGFP*/+. Note that although *ds^-^, ds^S>Ax3^-EGFP*, and *ds^-^ fj^-^, ds^S>Ax3^-EGFP* wings appear similar, in the absence of *fj* the planar polarity phenotype was sometimes slightly stronger, with swirls more often seen above vein 3. For quantification of this polarity phenotype, see Figure S3K.

To assay the activity of the transgenes, we looked at their effects on wing size, a parameter modulated by Fj, Ds, and Ft through their regulation of the Warts/Hippo pathway [1, 3]. Loss of *ds* activity did not much alter wing area (Figure 4F) but did affect wing shape (Figure 4B). However, uniform Ds expression reduces the area of the wing [5, 18–20], which we also see if we express *Act-ds-EGFP* uniformly in a *ds* background (Figures 4C and 4F). Notably, removal of *fj* from these *ds^−^, Act-ds-EGFP* wings reduced their size dramatically (Figures 4C′ and 4F; Figure S3B), even though removing *fj* from *ds*^−^ wings had only a minor effect (Figures 4B′ and 4F; Figure S3A). Hence, in the presence of *ds* activity, *fj* has a potent and easily measurable effect on wing size.

Importantly, the wings of *ds^−^, Act-ds^S>Ax3^-EGFP* flies (expressing the unphosphorylatable form of Ds) were indistinguishable in size and shape from those of *ds^−^ fj^−^, Act-ds-EGFP* flies (Figures 4C′, 4D, and 4F), and their size was not further altered if *fj* was removed (Figures 4D′ and 4F; Figure S3C). When uniformly expressed, the phosphomimetic form of Ds (Ds^S>Dx3^*-*EGFP) also affected wing area (Figures 4E and 4F) and was likewise insensitive to the presence of Fj (Figures 4E′ and 4F; Figure S3D). From these results, we conclude that the effects of Fj on wing size in this assay depend on the three key phosphorylation sites.

Recent data suggest that steeply graded Fj distribution and, consequently, steeply graded Ft-Ds activity promote growth [3, 18, 20]. And, consistent with this, overexpression of evenly expressed *fj*, which should tend to flatten a Ft-Ds gradient of activity, decreases wing size [5, 11, 18–20]. Further, the reduction in wing size we now see in *ds^−^ fj^−^, Act-ds-EGFP* wings is again consistent because Ds-EGFP is, presumably, evenly distributed. Overall, our results are consistent with the hypothesis that graded Fj activity (and hence graded Ft-Ds binding) is a determinant of wing size.

We note that Ds^S>Dx3^ might be expected to mimic the effect of uniform Fj on the wing, which reduces wing size [18, 20], and indeed, *ds^−^, ds^S>Dx3^-EGFP* wings are smaller than *ds^−^, ds-EGFP* wings (Figures 4C, 4E, and 4F). However, *ds^−^, ds^S>Dx3^-EGFP* wings are slightly larger than *ds^−^, ds^S>Ax3^-EGFP* wings (Figures 4D–4F; Figure S3E), which mimic the effect of loss of *fj* activity. A likely explanation for this difference in size is that in vivo, as in vitro, Ds^S>Dx3^ binds less strongly than Ds^S>Ax3^ to Ft, resulting in reduced Ft/Ds-mediated suppression of growth. Taken together, our data indicate that the primary effect of Fj on wing size is mediated by modulating the steepness of the Ft-Ds-binding gradient, with a smaller contribution attributable to the effect of Fj on overall strength of Ft-Ds binding.

We also studied planar polarity and found that our results argue that the same three phosphorylation sites in Ds are important for the planar polarity function of Fj. Ds-EGFP expression almost completely rescued the polarity defects seen in *ds^−^* wings (Figures 4G–4I; Figure S3G–S3I and S3K). Notably, in these wings (that lack graded *ds* expression), Fj activity made a contribution to planar polarity patterning, because its removal caused swirls (Figure 4H′; Figures S3H′ and S3K), indicating that, as in growth, Fj regulation of Ft-Ds binding is required for planar polarity. Both the *ds^−^, Act-ds^S>Ax3^-EGFP* and *ds^−^, Act-ds^S>Dx3^-EGFP* mutant wings show swirls around veins 3 and 4, similar to *ds^−^ fj^−^, Act-ds-EGFP* wings, consistent with a role for these phosphorylation sites in regulating planar polarity. However, in these genotypes, removal of *fj* causes a slight increase in the polarity phenotype, with swirls now seen above vein 3 (Figures 4J and 4J′; Figures S3J–S3K), indicating that in the context of planar polarity, Fj can act either via an alternative mechanism on Ds itself or via another molecule, for example Ft.

Overall, our results argue that Fj affects wing size via phosphorylation of Ds at the three identified residues. Furthermore, these sites also act to mediate effects of Fj on planar polarity.

### Conclusions

*fj* was first shown to interact with *ds* by Waddington [21], and subsequent studies showed that it regulates the functions of *ds* and *ft* [4, 6–9, 12]. Fj has recently been found to act in the Golgi [12] and mediate the phosphorylation of a subset of cadherin repeats of both Ft and Ds [13], but the function in vivo of this phosphorylation was not known. Here we have restricted our attentions largely to the action of Fj on Ds. We show that phosphorylation of Ds by Fj reduces the binding affinity of Ds for Ft, indicating that phosphorylation of Ds is important in the regulation of Ft/Ds heterodimerization and therefore of downstream consequences on growth and planar polarity. Furthermore, we find that, in vivo, a specific set of three phosphorylation sites in the Ds protein is required for Fj to mediate effects (via Ds) on the regulation of wing size and planar polarity. Our data additionally reveal that, in vivo, Fj also acts via Ft, even in the absence of Ds, although we have not found the mechanism. A possibility is that Fj phosphorylation of Ft promotes its binding to Ds, but we did not detect this in our assay. In this context, note that it might seem counterintuitive if phosphorylation of residues in Ft were to increase binding and if phosphorylation of analogous residues in Ds were to decrease binding. But, in any case, characterizing how Ft and Ds interact with each other is central to understanding how these giant cadherin molecules together regulate growth and polarity of tissues.

## Experimental Procedures

### Molecular Biology

Constructs were generated via standard molecular biology techniques, and mutagenized and polymerase chain reaction-amplified regions were verified by sequencing. *ds* and *ft* constructs were expressed under control of the *Act5C* promoter in both tissue culture experiments and transgenic flies. Full-length *ds* was created from *ds* cDNA fragments (gift from M. Noll) [22]. The untagged form lacks the final 303 aa because of a premature stop codon in the original cDNA, which was corrected in the Ds-EGFP form. Point mutations in the cadherin domains were introduced with QuikChange Multi Site-Directed Mutagenesis kit (Stratagene). Full-length *ft* was a 22 kb genomic fragment from BACR11D14, containing the entire coding sequence. Ft1-5 and Ds1-5 contained the first five cadherin repeats, followed by the complete transmembrane and cytoplasmic domains. Ds1-5sec and Ft1-5sec were truncated after the first five cadherin domains, and these constructs were C-terminally Myc-, HA-, GFP-, or RFP-tagged via the *Drosophila* Gateway system. *fj* constructs were expressed under upstream activating sequence (UAS) control [23] in transgenic flies, under the copper-inducible metallothionein promoter in the vector pMK33B for tissue culture experiments, and in the pENTR vector for the coimmunoprecipitation experiments in which they were additionally Myc tagged. The Golgi-tethered forms were made by swapping the appropriate region of the coding sequence into GNT-Fj [12].

### Fly Strains

Alleles used are described in FlyBase [24]. Loss-of-function clones that simultaneously express a protein under UAS control were generated by heat shocking third instar larvae for 1 hr at 37°C.

The following genotypes were used for *fj*, *ectoDs*, and *ft* expression in *ds ft* mutant clones:

y *w Scer\FLP1^hs.PS^ Scer\Gal4^alphaTub84B.PL^ Avic\GFP^Scer\UAS.T:Hsap\MYC,T:SV40\nls2^; ds^UA071^ ft^15^ stc P{FRT(w^hs^)}39*/ *Scer\GAL80^alphaTub84B.PL^ P{FRT(w^hs^)}39; fj^Scer\UAS.cZa^*/ *ft^Scer\UAS.cMa^*
y *w Scer\FLP1^hs.PS^ Scer\Gal4^alphaTub84B.PL^ Avic\GFP^Scer\UAS.T:Hsap\MYC,T:SV40\nls2^; ds^UA071^ ft^15^ stc P{FRT(w^hs^)}39*/ *Scer\GAL80^alphaTub84B.PL^ P{FRT(w^hs^)}39; MRS/ ft^Scer\UAS.cMa^*
y *w Scer\FLP1^hs.PS^ Scer\Gal4^alphaTub84B.PL^ Avic\GFP^Scer\UAS.T:Hsap\MYC,T:SV40\nls2^; ds^UA071^ ft^15^ stc P{FRT(w^hs^)}39*/ *Scer\GAL80^alphaTub84B.PL^ P{FRT(w^hs^)}39; fj^Scer\UAS.cZa^*/ *ds^ecto.Scer\UAS^*
y *w Scer\FLP1^hs.PS^ Scer\Gal4^alphaTub84B.PL^ Avic\GFP^Scer\UAS.T:Hsap\MYC,T:SV40\nls2^; ds^UA071^ ft^15^ stc P{FRT(w^hs^)}39*/ *Scer\GAL80^alphaTub84B.PL^ P{FRT(w^hs^)}39; MRS/ ds^ecto.Scer\UAS^*

The following genotype was used for *fj* expression in *fj* clones:

y *w Scer\FLP1^hs.PS^*; *P{neoFRT}42D pwn fj^d1^*/ *P{neoFRT}42D Scer\Gal80^alphaTub84B.PL^ Rnor\CD2^hs.PJ^*; *UAS.X*/ *Scer\Gal4^alphaTub84B.PL^*

where UAS.X means either a wild-type Fj sequence or one of the three mutant forms of Fj under UAS control. To make simple *fj*^−^ clones, we substituted UAS.X with a wild-type chromosome.

*ds-EGFP* and point mutants were subcloned into *pAttB-Act-FRT-polyA-FRT* (derived from *pAct-FRT-polyA-FRT* [25]), and transgenes were integrated into the same landing site (VK26 at 96F3 [26]) by BestGene. The following genotypes were used:

y *w Scer\FLP1^Ubx.hs^; ds^38k^ /ds^UA071^ ; AttB{w^+^ ActP-FRT-polyA-FRT-dsX-EGFP}/+*
y *w Scer\FLP1^Ubx.hs^; ds^38k^ fj^P1^/ ds^UA071^ fj^d1^; AttB{w^+^ ActP-FRT-polyA-FRT-dsX-EGFP}/+*

where *dsX* refers to wild-type *ds* or one of the *ds* point mutants.

### Histology and Antibodies

Abdominal cuticles were dissected and mounted in Hoyer's medium, and extended-focus images were generated with Helicon Focus (Heliconsoft). Adult wings were mounted in GMM. *Drosophila* S2 cells were fixed in 2% paraformaldehyde and washed in phosphate-buffered saline (PBS) 0.1% Triton X-100 prior to immunolabeling. Primary antibodies used for histology were rat anti-Fj [12], rabbit anti-Ds [6], chicken anti-GFP (Chemicon), mouse monoclonal anti-Fmi (Drosophila Studies Hybridoma Bank) (Usui et al. [28]), and a rat serum against the intracellular domain of Ft, which was generated with a His-tagged fusion protein corresponding to amino acids 4665–4859. Secondary antibodies used were anti-Rb Cy2, RRX and Cy5, anti-Chicken FITC (Jackson), anti-Rat A568, and anti-mouse A568 (Molecular Probes).

### Cell Culture

*Drosophila* S2 cells were grown in Schneider's medium (GIBCO) with fetal calf serum and transfected with Effectene (QIAGEN). *Drosophila* D.mel-2 cells were grown in ExpressFive SFM medium (Invitrogen) with L-glutamine and antibiotics and were transfected with Cellfectin (Invitrogen).

### Cell Aggregation Assay

For the cell aggregation assay, S2 cells were transfected with *pAct-ft* or *pAct-ds* (or *ds-EGFP*) along with *pMK33B* constructs expressing forms of *fj*. 2.5 × 10^5^ cells from the same transfection were split into separate wells of a nonadherent 24-well plate. CuSO_4_ was added to one well for 16 hr to induce Fj expression. 2.5 × 10^5^ cells singly transfected with *ft* or *ds* were added to the doubly transfected cells, rotated on a platform (100 rpm, 2 hr), transferred to a glass coverslip, and allowed to adhere for 2 hr, followed by immunolabeling of cell aggregates. Binding rates were determined by counting the percentage of *ft*-expressing cells in the population binding to *ds*-expressing cells (>200 Ft cells counted per coverslip; counting was done blind). Binding rate can vary depending on transfection frequency, so we compared the binding rates between cells from the same transfection but with one set of cells induced to express Fj. A reduction in binding was seen over a range of CuSO_4_ concentrations (0.07–0.7 mM) when Fj was expressed with Ds but not Ft. Graphs show averages of at least three separate experiments. FLAG-tagged Flamingo (Fmi) [27], a cadherin-containing protein that will bind homophilically [28], was used as a control in cell aggregation assays. Assays were also carried out to test interactions between tagged Ds1-5 and Ft1-5. Images are averages of three confocal sections taken on an Olympus FV1000 confocal microscope and processed in ImageJ and Photoshop.

### Cell Surface Staining

To stain for Ds specifically at the cell surface, we immunolabeled cells expressing Ds-EGFP in the absence of detergent with an anti-Ds primary antibody raised against the extracellular domain of the protein [6] followed by an RRX-conjugated secondary antibody. Cells were then labeled with anti-GFP and secondary antibodies in the presence of detergent to label total Ds. Images of cells were collected on the same confocal settings. Fluorescence intensity of cell surface Ds and total Ds was measured for individual cells with Volocity (Perkin Elmer). Forty to fifty cells were measured in each experiment and averaged. Student's t tests were performed on at least three separate experiments to test significance.

### Coimmunoprecipitations and Western Blotting

Transfected D.mel2 cells were harvested 24–48 hr after transfection, washed with cold PBS, and lysed (50 mM HEPES [pH 7.4], 100 mM NaCl, 0.5% NP40, 10% glycerol or 50 mM Tris-HCl [pH 7.4], 150 mM NaCl, 1 mM EDTA, and 1% Triton X-100, plus protease inhibitors). For western blots, rat or rabbit anti-Fj [12], mouse anti-GFP (Abcam), mouse anti α-tubulin DM1A (Sigma), mouse anti-Myc (Santa Cruz), mouse anti-HA (Roche), and secondaries conjugated to horseradish peroxidase (Dako) were used. Immunoprecipitations were carried out with mouse anti-Myc, and pulled-down proteins were detected by western blotting with anti-HA. For coimmunoprecipitation between Ds1-5sec and Ft1-5, media was collected from cells expressing Ds1-5sec after 48 hr and mixed with lysate from cells expressing Ft1-5. The cells expressing Ds1-5sec were also cotransfected with *pMK33B-GNT-fj* or kinase mutants to study the effect of phosphorylation of Ds on binding to Ft (because GNT-Fj is retained in the cell, it should not interfere with Ft-Ds binding in the lysate).

## Supplemental Information

Document S1. Three Figures
