# Supplementary material for: Four-Jointed Modulates Growth and Planar Polarity by Reducing the Affinity of Dachsous for Fat
Source: Curr Biol. 2010 May 11;20(9):803–10. doi: 10.1016/j.cub.2010.03.056 (PMC2958304; doi:10.1016/j.cub.2010.03.056)
Supplement: Document S1. Three Figures [file mmc1.pdf]

Report

Four-Jointed Modulates Growth and Planar Polarity by Reducing the Affinity of Dachsous for Fat

Amy L. Brittle,[1,4] Ada Repiso,[2,3,4,5] José Casal,[3,4] Peter A. Lawrence,[2,3,4,*] and David Strutt[1,4,**]

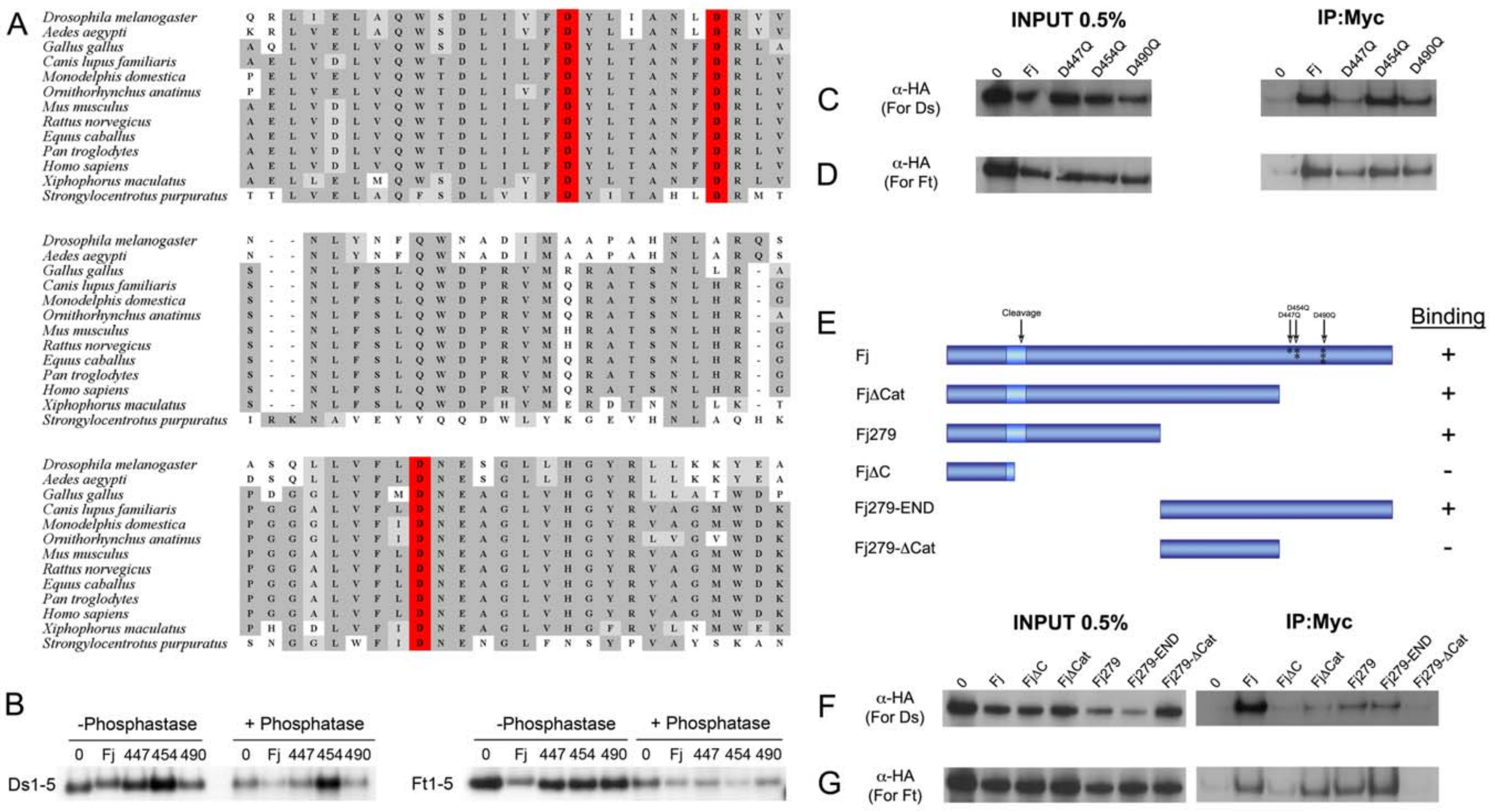
A
Drosophila melanogaster
Aedes aegypti
Gallus gallus
Canis lupus familiaris
Monodelphis domestica
Ornithorhynchus anatinus
Mus musculus
Rattus norvegicus
Equus caballus
Pan troglodytes
Homo sapiens
Xiphophorus maculatus
Strongylocentrotus purpuratus
B
-Phosphastase
+ Phosphatase
0 Fj 447 454 490
Ds1-5
Ft1-5
INPUT 0.5%
IP:Myc
0 Fj D447Q D454Q D490Q
C
α-HA (For Ds)
D
α-HA (For Ft)
E
Cleavage
D454Q
D447Q
D490Q
Binding
Fj +
FjΔCat +
Fj279 +
FjΔC -
Fj279-END +
Fj279-ΔCat -
INPUT 0.5%
IP:Myc
0 Fj FjΔC FjΔCat Fj279 Fj279-END Fj279-ΔCat
F
α-HA (For Ds)
G
α-HA (For Ft)

Figure S1, Related to Figure 1. Fj Has a Highly Conserved C-Terminal Kinase-Type Domain and Will Co-IP with Truncated Forms of Ft and Ds

(A) Alignment of amino acids 432 to 508 of *Drosophila* Fj protein with other species. In red are shown the conserved aspartate (D) residues that were mutagenised in this study.

(B) Truncated forms of Ds and Ft (Ds1-5sec-HA and Ft1-5sec-HA), consisting of the first five cadherin repeats, show reduced mobility on western blots indicative of modification by phosphorylation [13] when coexpressed with wildtype Fj in D.mel2 cells but not when coexpressed with the three kinase mutants. This mobility shift is reversed by treatment with phosphatase.

(C,D) Co-IPs of wildtype and mutated Myc-tagged Fj with Ds1-5-HA and Ft1-5-HA. The control without Fj protein is shown as 0. Ds (A) and Ft (B) co-IP with both wildtype Fj and the kinase domain mutants, although D447Q shows reduced binding.

(E) Diagrams of Fj constructs used for co-IPs. Ability to bind to Ds1-5 and Ft1-5 is indicated on right.

(F,G) Co-IPs of wildtype and truncated forms of Myc-tagged Fj with Ds1-5-HA and Ft1-5-HA, to map the region of interaction. Both Ds1-5 and Ft1-5 interact with full-length Fj, FjΔCat, Fj279 and Fj279-END but not Fj279-ΔCat or FjΔC, indicating the presence of binding regions within Fj in both the catalytic kinase domain and more N-terminally.

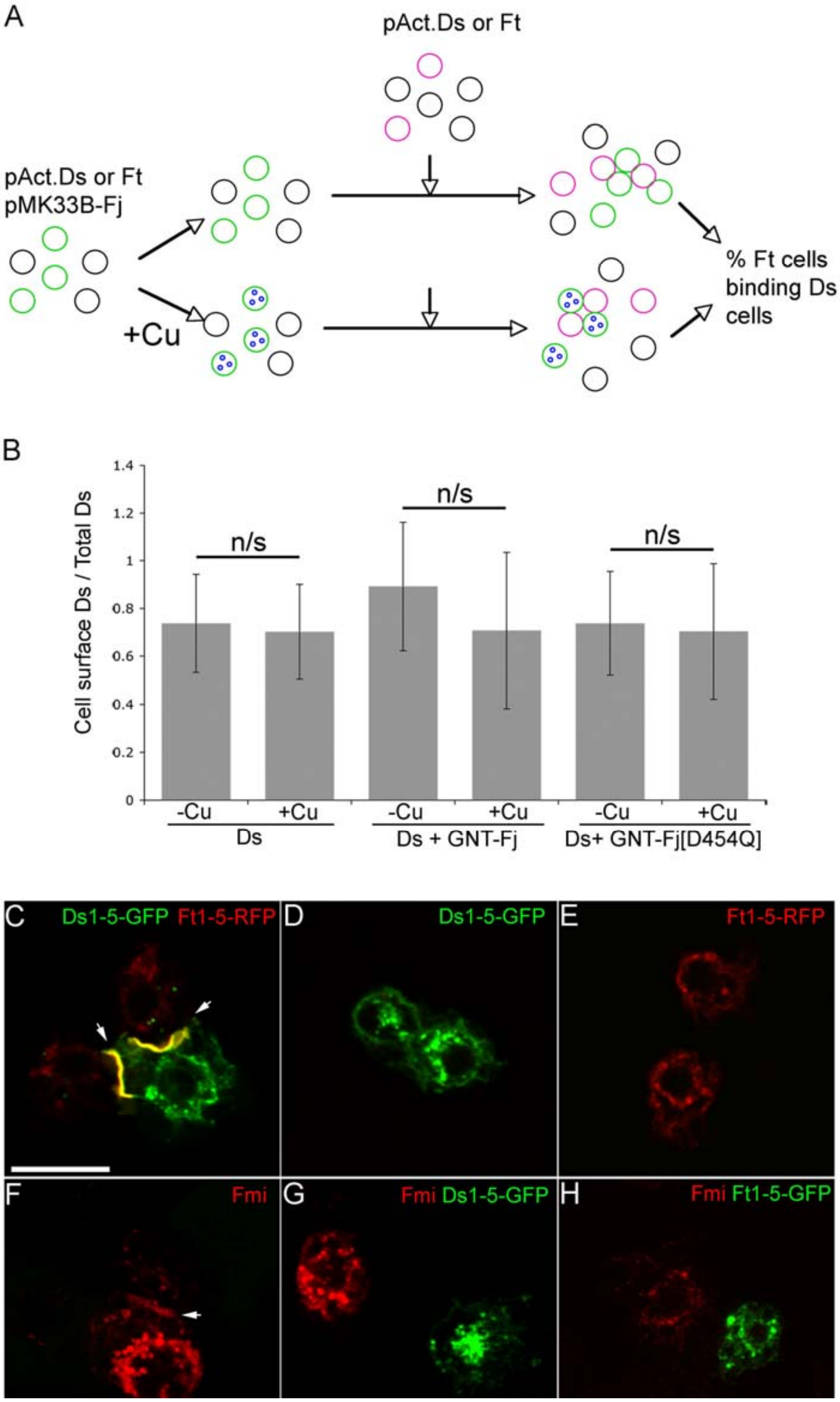

A
pAct.Ds or Ft
pAct.Ds or Ft
pMK33B-Fj
+Cu
% Ft cells
binding Ds
cells
B
Cell surface Ds / Total Ds
1.4
1.2
1
0.8
0.6
0.4
0.2
0
n/s
n/s
n/s
-Cu
+Cu
-Cu
+Cu
-Cu
+Cu
Ds
Ds + GNT-Fj
Ds+ GNT-Fj[D454Q]
C
Ds1-5-GFP Ft1-5-RFP
D
Ds1-5-GFP
E
Ft1-5-RFP
F
Fmi
G
Fmi Ds1-5-GFP
H
Fmi Ft1-5-GFP

Figure S2, Related to Figure 2. Scheme of Cell Aggregation Assay, Level of Cell Surface Ds-EGFP in the Presence and Absence of GNT-Fj and GNT-Fj^{D454Q} and Truncated Ft and Ds Binding in Cell Aggregation Assays

(A) Drosophila S2 cells were doubly transfected with *pAct-ds* or *pAct-ft* and *pMK33B-fj* (copper-inducible) (green cells). Cells from the same transfection, and therefore expressing Ft or Ds at the same level, were split into 2 wells of a 24 well tissue culture plate, and $CuSO_4$ was added to one well to induce expression of Fj. Doubly transfected cells were then mixed with cells singly transfected with *pAct-ft* or *pAct-ds* (magenta cells) and rotated on a platform, before being allowed to adhere to a coverslip and immunolabelled. Ft and Ds expressing cells bind to form cell aggregates. The level of binding was then quantified by counting the percentage of Ft cells binding Ds cells. To determine the effect of *fj* expression, the rates of binding in cells minus and plus Fj were compared.

(B) Fluorescence intensity of cell surface Ds-EGFP and total Ds-EGFP was measured for 40-50 individual cells and the values shown as a ratio. Values shown on the graph are the mean of 5 different experiments, errors bars show standard deviation. Student t-tests were performed and no significant differences were found.

(C-H) Confocal images showing protein distribution revealed by immunofluorescence of S2 cell expressing cadherin proteins. Arrows point to stabilisation of protein at the cell interface in cell aggregates. Scale bar is 10µm. All images are the same magnification. Truncated forms of Ds and Ft will bind and stabilise each others' localisation at the cell interface but will not bind to themselves or to another cadherin, Flamingo (Fmi, also known as Starry Night).

(C) Ds1-5-GFP and Ft1-5-RFP

(D) Ds1-5-GFP

(E) Ft1-5-RFP

(F) Fmi-FLAG will bind to itself in neighbouring cells.

(G) Fmi-FLAG and Ds1-5-GFP

(H) Fmi-FLAG and Ft1-5-GFP

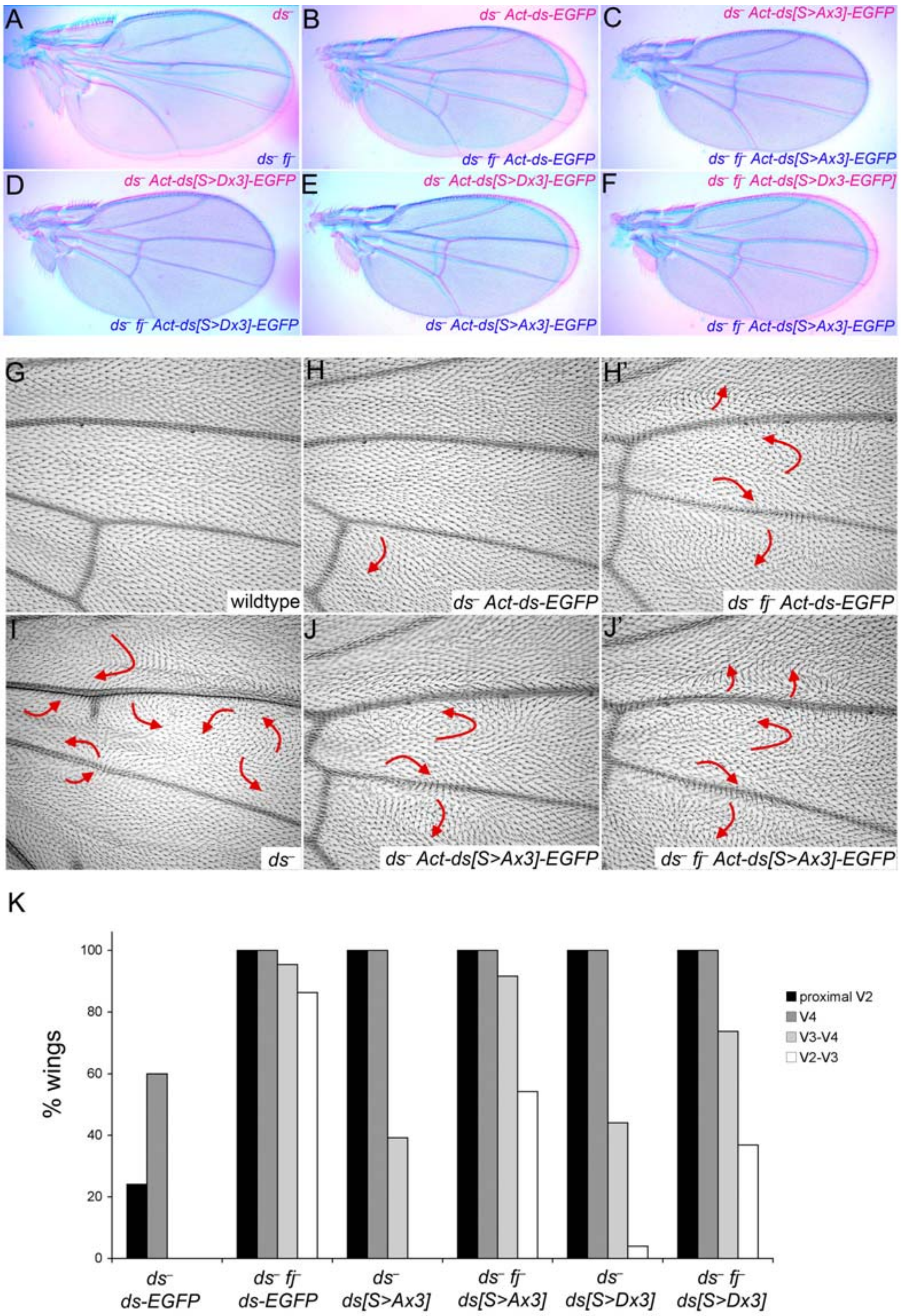
A
ds–
ds– fj–
B
ds– Act-ds-EGFP
ds– fj– Act-ds-EGFP
C
ds– Act-ds[S>Ax3]-EGFP
ds– fj– Act-ds[S>Ax3]-EGFP
D
ds– Act-ds[S>Dx3]-EGFP
ds– fj– Act-ds[S>Dx3]-EGFP
E
ds– Act-ds[S>Dx3]-EGFP
ds– Act-ds[S>Ax3]-EGFP
F
ds– fj– Act-ds[S>Dx3-EGFP]
ds– fj– Act-ds[S>Ax3]-EGFP
G
wildtype
H
ds– Act-ds-EGFP
H'
ds– fj– Act-ds-EGFP
I
ds–
J
ds– Act-ds[S>Ax3]-EGFP
J'
ds– fj– Act-ds[S>Ax3]-EGFP
K
% wings
100
80
60
40
20
0
proximal V2
V4
V3-V4
V2-V3
ds– ds-EGFP
ds– fj– ds-EGFP
ds– ds[S>Ax3]
ds– fj– ds[S>Ax3]
ds– ds[S>Dx3]
ds– fj– ds[S>Dx3]

Figure S3, Related to Figure 4. Ds Phosphorylation Sites Are Required In Vivo for Fj to Modulate Growth and Planar Polarity

(A-F') Overlays of male adult wings of some of the genotypes shown in Fig.4. All images are of the same magnification.

(A) ds^{UA071}/ds^{38k} and ds^{UA071} fj^{d1}/ds^{38k} fj^{P1}

(B) ds^{UA071}/ds^{38k}*; Act-ds-EGFP/+* and ds^{UA071} fj^{d1}/ds^{38k} fj^{P1}*; Act-ds-EGFP/+*

(C) ds^{UA071}/ds^{38k}*; Act-*$ds^{S>Ax3}$*-EGFP/+* and ds^{UA071} fj^{d1}/ds^{38k} fj^{P1}*; Act-*$ds^{S>Ax3}$*-EGFP/+*

(D) ds^{UA071}/ds^{38k}*; Act-*$ds^{S>Dx3}$*-EGFP/+* and ds^{UA071} fj^{d1}/ds^{38k} fj^{P1}*; Act-*$ds^{S>Dx3}$*-EGFP/+*

(E) ds^{UA071}/ds^{38k}*; Act-*$ds^{S>Ax3}$*-EGFP/+* and ds^{UA071}/ds^{38k}*; Act-*$ds^{S>Dx3}$*-EGFP/+*

(F) ds^{UA071} fj^{d1} $/ds^{38k}$ fj^{P1}*; Act-*$ds^{S>Ax3}$*-EGFP/+*and ds^{UA071} fj^{d1}/ds^{38k} fj^{P1}*; Act-*$ds^{S>Dx3}$*-EGFP/+*

(G-J') Representative images of the planar polarity of hairs on the ventral surface in adult wings. Arrows mark changes in the direction hairs point. Note that distally pointing hairs on the dorsal surface of the wing can also be seen in a deeper plane of focus. Polarity swirls on the dorsal surface were significantly weaker than on the ventral surface.

(G) wildtype

(H) ds^{UA071}/ds^{38k}*; Act-ds-EGFP/+.*

(H') ds^{UA071} fj^{d1}/ds^{38k} fj^{P1}*; Act-ds-EGFP/+*

(I) ds^{UA071}/ds^{38k}

(J) ds^{UA071}/ds^{38k}*; Act-*$ds^{S>Ax3}$*-EGFP/+*

(J') ds^{UA071} fj^{d1}/ds^{38k} fj^{P1}*; Act-*$ds^{\ S>Ax3}$*-EGFP/+.*

(K) Quantitation of hair swirls on the ventral surface of wings of the indicated genotypes. At least 20 wings from male flies were scored per genotype. Defects in planar polarity are scored by the presence of hair swirls in the indicated regions of the wing, according to the following key:

Proximal V2 - above vein 2, in the proximal region of the wing.

V4 - below vein 4, distal to the posterior cross-vein.

V3-V4 - between veins 3 and 4, distal to the posterior cross-vein.

V2-V3 - between veins 2 and 3, distal to the posterior cross-vein.
